# Inhibition of thioredoxin 1 leads to apoptosis in drug-resistant multiple myeloma

**DOI:** 10.18632/oncotarget.3795

**Published:** 2015-04-12

**Authors:** Prahlad V. Raninga, Giovanna Di Trapani, Slavica Vuckovic, Maneet Bhatia, Kathryn F. Tonissen

**Affiliations:** ^1^ School of Natural Sciences, Griffith University, Nathan, QLD, Australia; ^2^ Eskitis Institute for Drug Discovery, Griffith University, Nathan, QLD, Australia; ^3^ QIMR Berghofer Medical Research Institute, Brisbane, QLD, Australia

**Keywords:** thioredoxin, multiple myeloma, apoptosis, NF-kB, chemoresistance

## Abstract

Multiple myeloma (MM) is a hematological malignancy characterized by the aberrant accumulation of clonal plasma cells in the bone marrow. Despite recent advancement in anti-myeloma treatment, MM remains an incurable disease. This study showed higher intrinsic oxidative stress and higher Trx1 and TrxR1 protein levels in MM cells compared to normal cells. Drug-induced Trx1 (PX-12) and TrxR1 (Auranofin) inhibition disrupted redox homeostasis resulting in ROS-induced apoptosis in MM cells and a reduction in clonogenic activity. Knockdown of either Trx1 or TrxR1 reduced MM cell viability. Trx1 inhibition by PX-12 sensitized MM cells to undergo apoptosis in response to the NF-кβ inhibitors, BAY 11-7082 and curcumin. PX-12 treatment decreased the expression of the NF-кβ subunit p65 in MM cells. Bortezomib-resistant MM cells contained higher Trx1 protein levels compared to the parental cells and PX-12 treatment resulted in apoptosis. Thus, increased Trx1 enhances MM cell growth and survival and exerts resistance to NF-кβ inhibitors. Therefore inhibiting the thioredoxin system may be an effective therapeutic strategy to treat newly diagnosed as well as relapsed/refractory MM.

## INTRODUCTION

Multiple myeloma (MM) is a cancer characterized by aberrant accumulation of malignant monoclonal plasma cells in the bone marrow. MM accounts for 2% of all neoplasms and is the second most common form of hematological malignancies [1]. There are a number of chemotherapeutic agents that are being used as anti-myeloma therapies, for example, the immunomodulator thalidomide [2] and its derivative lenalidomide [3], and a proteasome inhibitor bortezomib [4]. Despite such advancements in anti-myeloma therapies, MM remains an incurable disease with only a slight increase in the median survival rate to 5 years [5]. Upon prolonged treatment MM cells acquire resistance to the given treatment, and thus relapse is inevitable. Therefore, novel therapeutic approaches to treat MM are desperately needed.

Intrinsic oxidative stress is a hallmark of cancer that give rise to the conditions leading to tumor development and progression [6]. In response to increased reactive oxygen species (ROS) levels and their toxic effects, cells have evolved a number of survival pathways, which includes the expression of antioxidant molecules and stress-response molecules. However, cancer cells exploit this advantage and adapt to the enhanced oxidative stress through an increased antioxidant capacity. Cancer cells with higher intrinsic oxidative stress are more resistant to the disruption of redox regulation compared to non-cancerous cells [7].

The thioredoxin (Trx) system is one of the major antioxidant systems that maintain the intracellular redox homeostasis [8]. The Trx system is comprised of thioredoxin 1 (Trx1), a cellular redox protein, thioredoxin reductase 1 (TrxR1) enzyme, and NADPH. The Trx system maintains cellular redox homeostasis by either directly scavenging ROS [9] or by regulating several redox enzymes [10, 11]. In addition, Trx1 also regulates various redox-sensitive transcription factors by acting as a co-factor to reduce specific cysteine residues [12, 13]. Trx1 also acts as a growth factor that stimulates cancer cell proliferation and tumor growth and inhibits spontaneous and drug-induced apoptosis [14]. Expression of Trx1 is upregulated in many human cancer types, correlating with cancer cell proliferation, survival, and chemoresistance [15-17]. Similarly, expression and activity of TrxR is upregulated in many human cancer types [15, 18] and its inhibition results in cancer cell apoptosis [19]. However, there are no previous reports on the physiological and therapeutic significance of the Trx family members in MM and its survival.

Nuclear factor-кβ (NF-кβ) is a nuclear transcription factor regulating the expression of various genes involved in cell proliferation, tumorigenesis, and inflammation. The NF-кβ family is comprised of five members including p50/p105 (NF-кβ1), p52/p100 (NF-кβ2), p65 (Rel A), c-Rel, and Rel B [20]. Under non-stimulating conditions, NF-кβ binds a class of inhibitor proteins called Inhibitor of кβ (Iкβ) [20]. Upon stimulation, Iкβ undergoes phosphorylation and subsequent degradation by the proteasome releasing NF-кβ, which then translocates into the nucleus where it activates gene expression [21]. NF-кβ and its signaling pathways are constitutively activated in MM cell lines as well as in patient myeloma cells [22] and constitutive NF-кβ activity is required for the pathogenesis of MM [23, 24]. While the proteasome inhibitor bortezomib inhibits NF-кβ signaling by preventing the degradation of Iкβ, resulting in MM cell apoptosis [25, 26], and significant improvement in terms of disease control and patient survival [4, 27], upon prolonged treatment myeloma cells become resistant to bortezomib [28].

Being a critical redox regulator, Trx1 activates several redox sensitive transcription factors including NF-кβ. Under oxidative stress conditions, Trx1 increases the DNA binding activity of NF-кβ subunits, enhancing NF-кβ mediated gene expression leading to cell proliferation and survival [29]. However, not much is known about the interaction between Trx1 and the NF-кβ pathway in MM cells.

The present study was designed to characterize the role of Trx1 and TrxR1 in myeloma cell survival, growth, and chemo-resistance. Our results show that higher Trx1 and TrxR1 expression levels correlate with myeloma cell survival and growth, and protect MM cells against increased intrinsic oxidative stress. Trx1 and TrxR1 inhibition results in ROS-induced apoptosis in MM cells. Our data also demonstrate that inhibition of Trx1 using a sub-lethal concentration of PX-12 sensitizes MM cells to undergo cell death in response to NF-кβ inhibition. Results show that Trx1 inhibition decreases levels of the NF-кβ subunit p65 protein in MM cells. Moreover, the Trx1 inhibiting drug PX-12 induces apoptosis of bortezomib-resistant MM cells. Thus, Trx1 may be an effective therapeutic target either alone or in combination with other NF-кβ targeted therapies in the treatment of newly diagnosed as well as relapse/refractory MM patients.

## RESULTS

### Multiple myeloma cells have higher intrinsic oxidative stress and an up-regulated thioredoxin system

**Figure 1:**
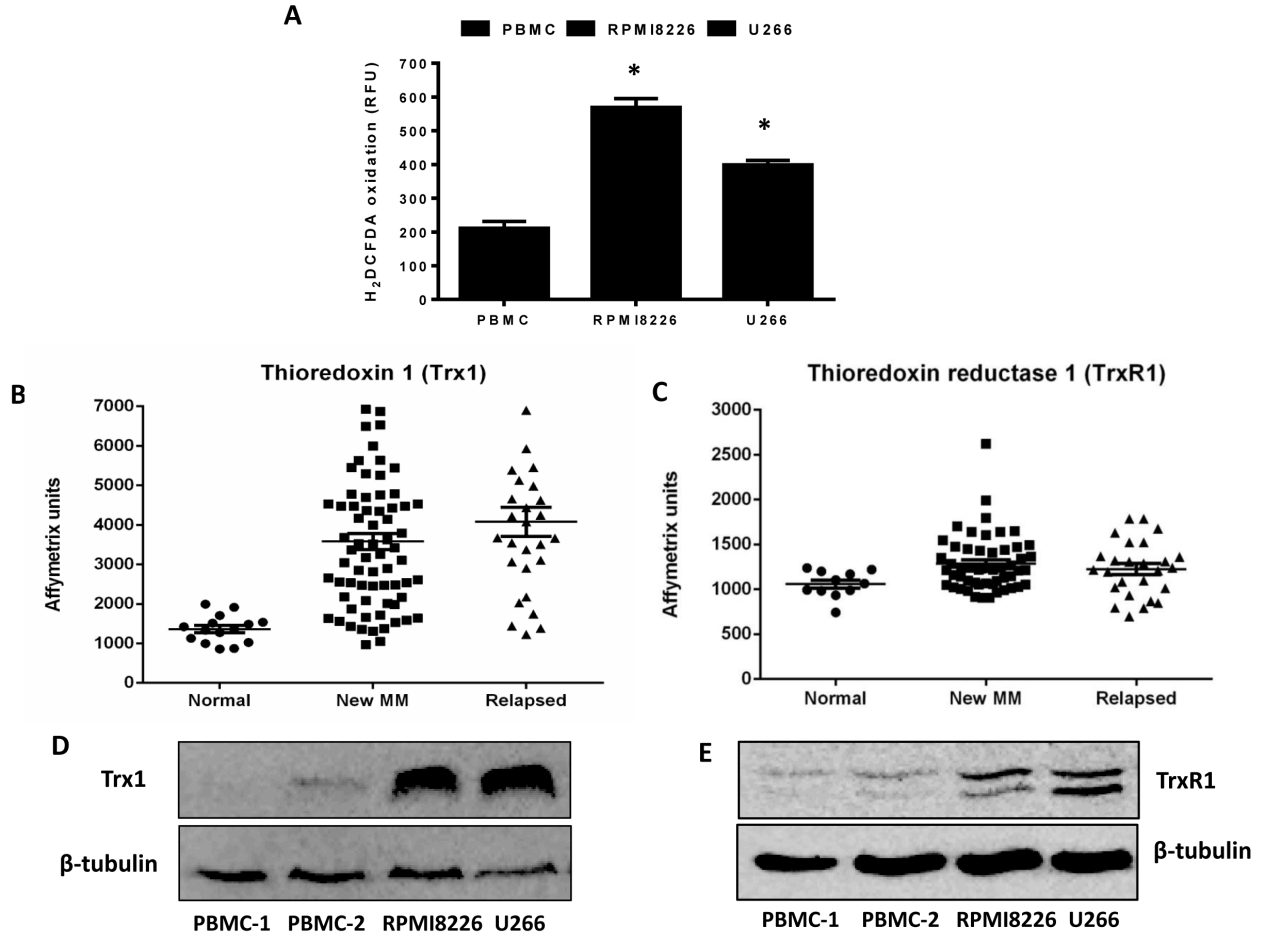
Multiple myeloma cells have higher intrinsic oxidative stress and an upregulated thioredoxin system **A.** Basal ROS levels were measured in myeloma cell lines (RPMI8226, U266) and two samples of control PBMCs using H_2_DCFDA. Values indicate mean ± SEM of three independent experiments performed in triplicate. One-way ANOVA followed by Tukey's post-test were employed. *, *P* < 0.05 (compared to PBMCs) **B. C.** Expression of Trx1 and TrxR1 in patient myeloma cells (new MM and relapsed) and normal cells were determined from the gene expression profile arrays deposited in the gene expression omnibus database (GSE6477). One-way ANOVA followed by Tukey's post-test was performed. *P* < 0.001 (Trx1) and *P* < 0.05 (TrxR1) compared to normal cells. **D. E.** Whole cell extracts were prepared from myeloma cell lines (RPMI8226, U266) and control PBMCs, and Western blot analysis was conducted for Trx1 **D.** and TrxR1 **E.** protein levels. β-tubulin was used to confirm the equal loading. Western blots are representative of three independent experiments.

Increased oxidative stress is a hallmark of many human cancer types, which leads to aberrant cancer growth and proliferation [6]. The levels of intrinsic oxidative stress were examined in MM cell lines (RPMI8226, U266) and normal peripheral blood mononuclear cells (PBMCs) by analyzing the intracellular ROS levels. Both MM cell lines have significantly higher ROS levels compared to PBMCs (Figure 1A).

To examine whether MM cells have increased antioxidant capacity, we first evaluated the expression levels of Trx1 and TrxR1 in myeloma cells compared to PBMCs. A gene expression omnibus dataset (GSE6477) shows that both Trx1 (Figure 1B) and TrxR1 (Figure 1C) are expressed at significantly higher levels in new and relapsed myeloma patient cells compared to normal cells. Western blot analysis confirmed higher protein levels of Trx1 (Figure 1D) and TrxR1 (Figure 1E) in MM cell lines compared to PBMCs.

### Trx1 and TrxR1 inhibition reduces MM cell proliferation and viability

**Figure 2:**
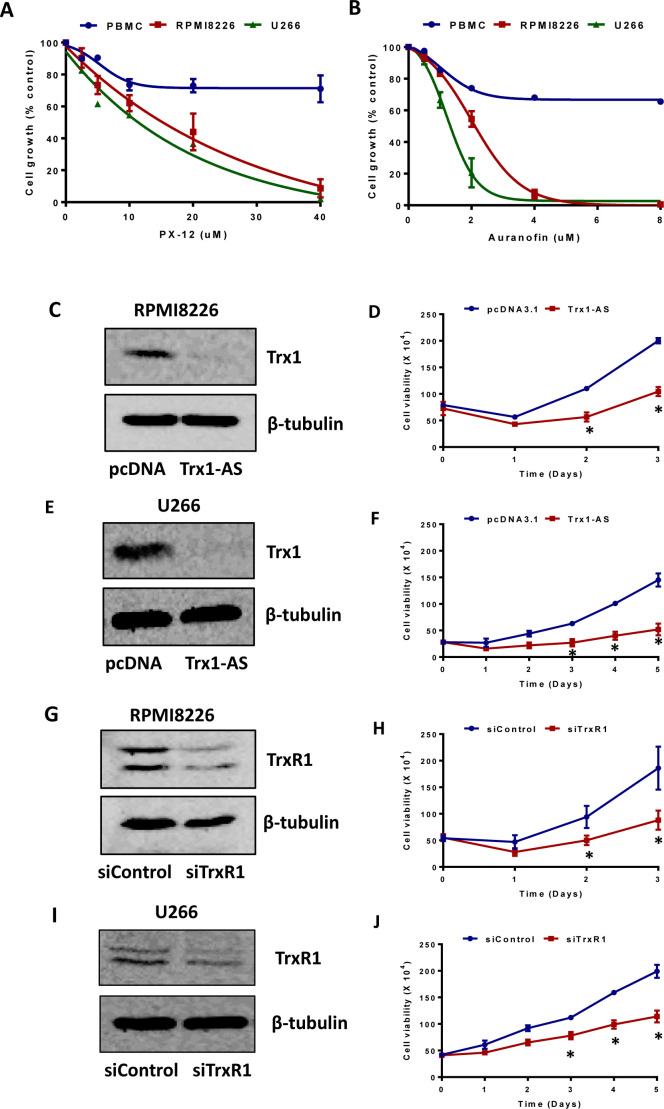
Inhibition of Trx1 and TrxR1 reduces myeloma cell proliferation and viability **A. B.** RPMI 8226, U266, and control PBMCs were treated with indicated concentrations of PX-12 **A.** and auranofin **B.** for 24 hours. Cell proliferation was assessed by MTT assays. Values indicate mean ± SEM of three independent experiments performed in triplicate. **C**. **D. E. F.** RPMI8226 **C. D.** and U266 **E. F.** cells were transfected with 2 μg of pcDNA 3.1 vector or Trx1-AS plasmid. Trx1 protein levels (24 hours) were analyzed by western blot in RPMI8226 **C.** and U266 **E.**. β-tubulin was used as a loading control. Cell viability was measured at the indicated time points by using Trypan blue exclusion method in RPMI8226 **D.** and U266 **F.**. **G. H. I. J.** RPMI8226 **G. H.** and U266 **I. J.** cells were transfected 30 nmol/L of either control or TrxR1 specific siRNA. TrxR1 protein levels (48 hours) were analyzed by western blot in RPMI8226 **G.** and U266 **I.**. β-tubulin was used as a loading control. Cell viability was measured at the indicated time points by using the Trypan blue exclusion method in RPMI8226 **H.** and U266 **J.**. Values indicate mean ± SEM (*n* = 3). Two-way ANOVA followed by Sidak's post-test was employed. *, *P* < 0.05.

To study the role of Trx1 and TrxR1 in the growth and survival of MM cells, we used both chemical inhibition and a knockdown approach. Auranofin reacts with selenol-containing residues present in TrxR1, inhibits its activity [30], and shows excellent anti-tumor activity [19]. PX-12 inhibits Trx1 by irreversibly alkylating the Cys73 residue [31] and has been shown to exert anti-tumor activity [32, 33]. We used PX-12 and auranofin as tools to study the cytoprotective functions of Trx1 and TrxR1 in MM cells. Treatment of RPMI8226, U266, and control PBMCs with increasing concentrations of PX-12 (0-40 μM) (Figure 2A) and auranofin (0-8 μM) (Figure 2B) for 24 hours resulted in a marked inhibition of RPMI8226 and U266 cell proliferation compared to PBMCs.

To ascertain if specific knock-down of Trx1 and TrxR1 could reproduce the effect of drug-induced Trx1 and TrxR1 inhibition on MM growth, we used the Trx1-antisense (Trx1-AS) plasmid DNA and TrxR1 specific siRNA. Transfection of the Trx1-antisense plasmid decreased Trx1 protein levels compared to the control vector (Figure 2C and 2E) and reduced RPMI8226 (Figure 2D) and U266 (Figure 2F) cell viability by 50% after 2 and 3 days of incubation, respectively. Similarly, siRNA against TrxR1 suppressed TrxR1 protein expression compared to the control siRNA (Figure 2G and 2I) and reduced RPMI8226 (Figure 2H) and U266 (Figure 2J) cell viability by approximately 30-50% after 2 and 3 days of incubation, respectively.

### Inhibition of Trx1 or TrxR1 decreases the clonogenic activity of MM cells

**Figure 3:**
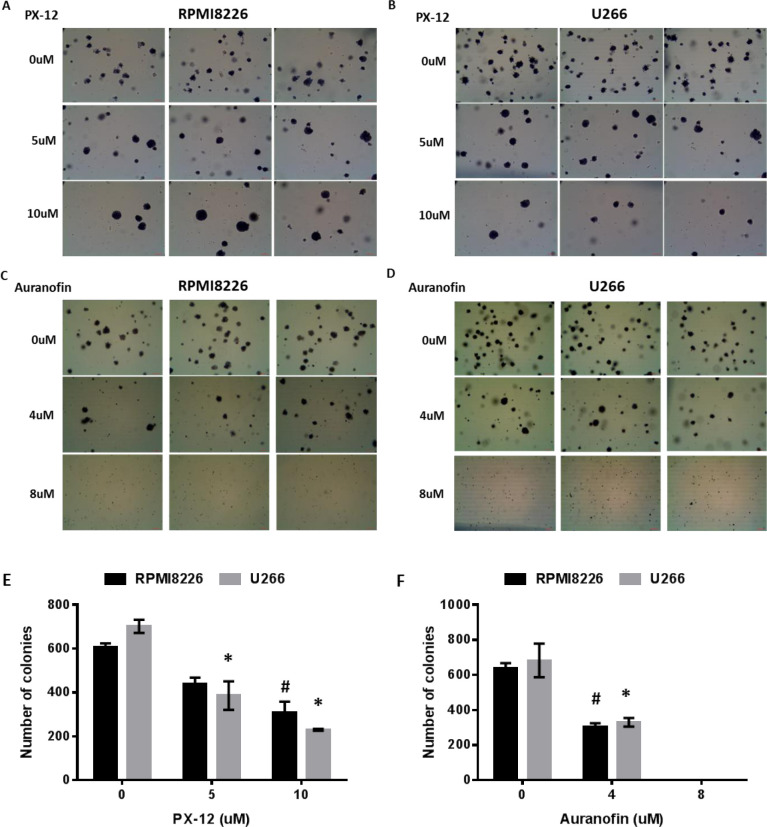
Inhibition of Trx1 or TrxR1 decreases clonogenic activity of MM cells **A. B.** Methylcellulose colony formation assay of RPMI8226 **A.** and U266 **B.** cells treated with 5 μM and 10 μM PX-12. **C. D.** Methylcellulose colony formation assay of RPMI8226 **C.** and U266 **D.** cells treated with 4 μM and 8 μM auranofin. **E.** Number of colonies formed after PX-12 treatment of RPMI8226 and U266 cells. **F.** Number of colonies formed after auranofin treatment of RPMI8226 and U266 cells. Values indicate mean ± SEM of three independent experiments performed in duplicate. One-way ANOVA followed by Tukey's post-test was employed. * (U266), # (RPMI8226), *P* < 0.05.

Myeloma cells have clonogenic activity and these clonogenic myeloma cells are resistant to a number of clinically used anti-myeloma drugs [34]. We investigated whether inhibiting either Trx1 or TrxR1 reduces clonogenic activity of myeloma cells. A significant reduction in the number of colonies was observed at 10 μM PX-12 in RPMI8226 (Figure 3A and 3E) and 5 μM PX-12 in U266 (Figure 3B and 3E). Similarly, 4 μM auranofin treatment significantly decreased the number of colonies in both MM cell lines (Figure 3C, 3D and 3F). No colonies were observed in MM cells treated with 8 μM auranofin. No significant effect on colony number was observed with lower concentrations of PX-12 or auranofin (data not shown).

### Inhibition of Trx1 or TrxR1 induces MM cell apoptosis

**Figure 4:**
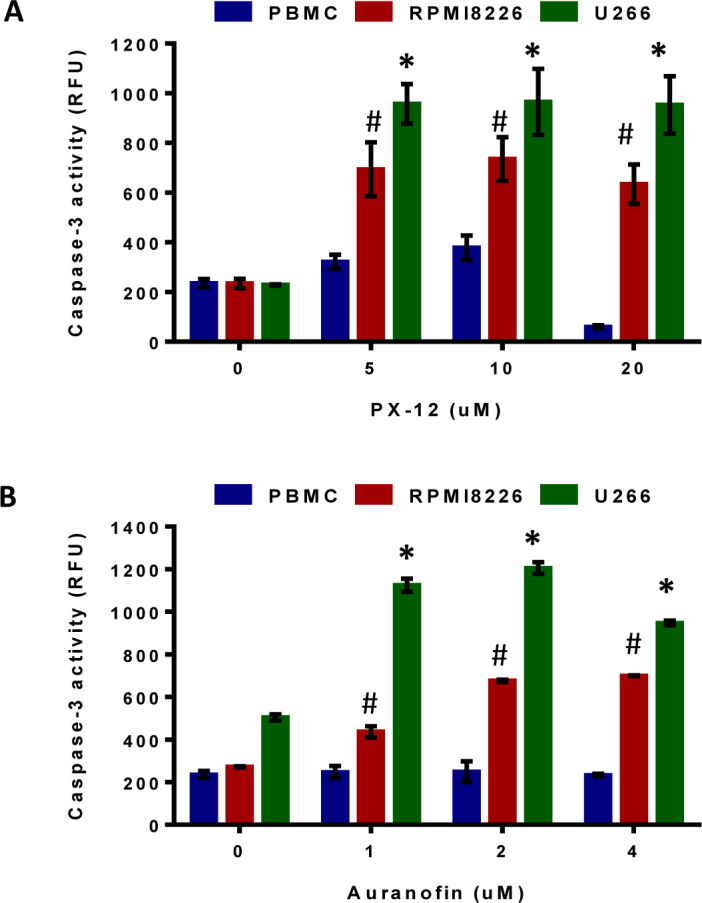
Inhibition of Trx1 or TrxR1 induces MM cell apoptosis **A, B**, RPMI8226, U266, and control PBMCs were treated with indicated concentrations of PX-12 (A) and auranofin (**B**) for 24 hours. Caspase-3 activity in treated and untreated myeloma cell lines and PBMCs was measured by monitoring the cleavage of Ac-DEVD-AMC. Values indicate mean ± SEM (*n* = 3). One-way ANOVA followed by Tukey's post-test was employed. * (U266), # (RPMI 8226), *P* < 0.0001.

Next we determined the mode of myeloma cell death in response to Trx1 and TrxR1 inhibition using specific inhibitors. RPMI8226, U266, and control PBMCs were treated with PX-12 (0-20 μM) and auranofin (0-4 μM) for 24 hours and caspase-3 activity was measured. 5 μM PX-12 significantly increased caspase-3 activity by 3-fold in RPMI8226 and 4-fold in U266 cells compared to non-treated cells (Figure 4A). Similarly, 2 μM auranofin significantly increased caspase-3 activity by 2.5-fold in RPMI8226 and 2.3-fold in U266 cells compared to non-treated cells (Figure 4B). No significant increase in caspase-3 activity was observed in PX-12 and auranofin-treated control PBMCs.

### Inhibition of the Trx system results in ROS-induced apoptosis in MM

**Figure 5:**
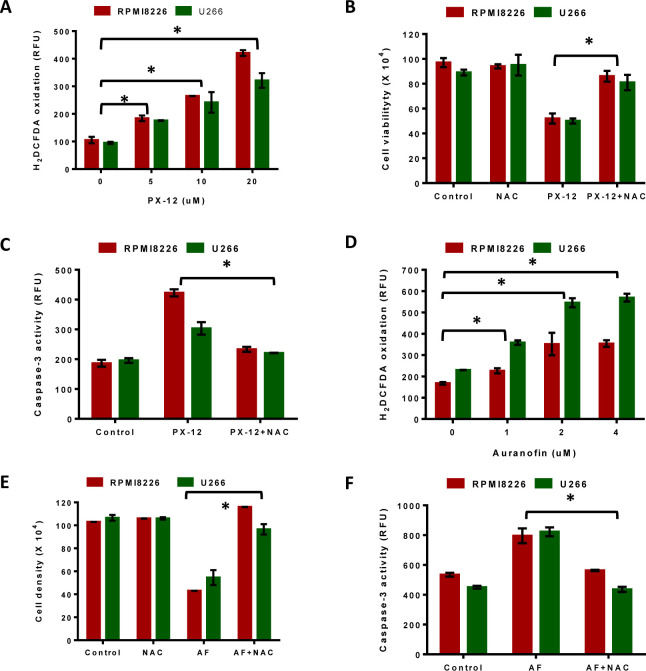
Inhibition of Trx system results in ROS-induced apoptosis in MM **A.** RPMI8226 and U266 cells were treated with indicated concentrations of PX-12 for 24 hours and then assessed for H_2_DCFDA oxidation. **B. C.** RPMI8226 and U266 cells were treated with 5 μM PX-12 alone or in combination with 5 mM NAC for 24 hours followed by measuring cell viability using Trypan blue exclusion **B.**, and examination of apoptosis by measuring Caspase-3 activity **C.**. **D.** RPMI8226 and U266 cells were treated with indicated concentrations of auranofin for 24 hours and then assessed for H_2_DCFDA oxidation. **E. F.** RPMI8226 and U266 cells were treated with 2 μM auranofin alone or in combination with 5 mM NAC for 24 hours followed by measuring cell viability using Trypan blue exclusion **E.** and examination of apoptosis by measuring caspase-3 activity **F.**. One-way ANOVA followed by Tukey's post-test was employed. *, *P* < 0.05. Values indicate mean ± SEM of three independent experiments performed in triplicate. For caspase-3 activity assay (*n* = 3).

We next investigated the role of Trx1 and TrxR1 in protecting MM cells against higher intrinsic oxidative stress and whether their inhibition disrupts intracellular redox homeostasis in myeloma cells. Treatment of RPMI8226 and U266 cells with PX-12 (0-20 μM) for 24 hours showed a significant increase in the amount of ROS at 5 μM PX-12 as indicated by H_2_DCFDA oxidation (Figure 5A). To investigate the involvement of ROS, we analyzed whether two antioxidants, N-acetyl cysteine (NAC) and ascorbic acid, protected MM cells against PX-12-induced apoptosis. Treatment of both cell lines with 5 μM PX-12, with or without 5 mM NAC, showed that addition of NAC significantly abrogated the PX-12-induced increase in ROS in both MM cell lines (Figure S1). These results confirmed that Trx1 inhibition by PX-12 increases intracellular ROS levels in MM. Moreover, treatment of cells with NAC (5 mM) together with PX-12 (5 μM) significantly rescued MM cells from undergoing cell death (Figure 5B) and also abrogated PX-12-induced apoptosis in both myeloma cell lines (Figure 5C). Ascorbic acid (AA) showed a similar protective effect, but was not as effective as NAC (Figure S2).

We then investigated the effect of auranofin on the intracellular ROS levels in MM cells. Treatment of RPMI8226 and U266 cells with auranofin (0-4 μM) significantly increased intracellular ROS levels in both MM cell lines (Figure 5D). In addition, addition of 5 mM NAC to auranofin-treated RPMI8226 and U266 cells significantly rescued them from undergoing cell death (Figure 5E) and apoptosis (Figure 5F). Addition of ascorbic acid also showed a similar protective effect, but was observed to be less effective than NAC (Figure S2).

### Trx1 inhibition sensitizes myeloma cells to NF-кβ inhibitors and decreases NF-кβ p65 protein expression

**Figure 6:**
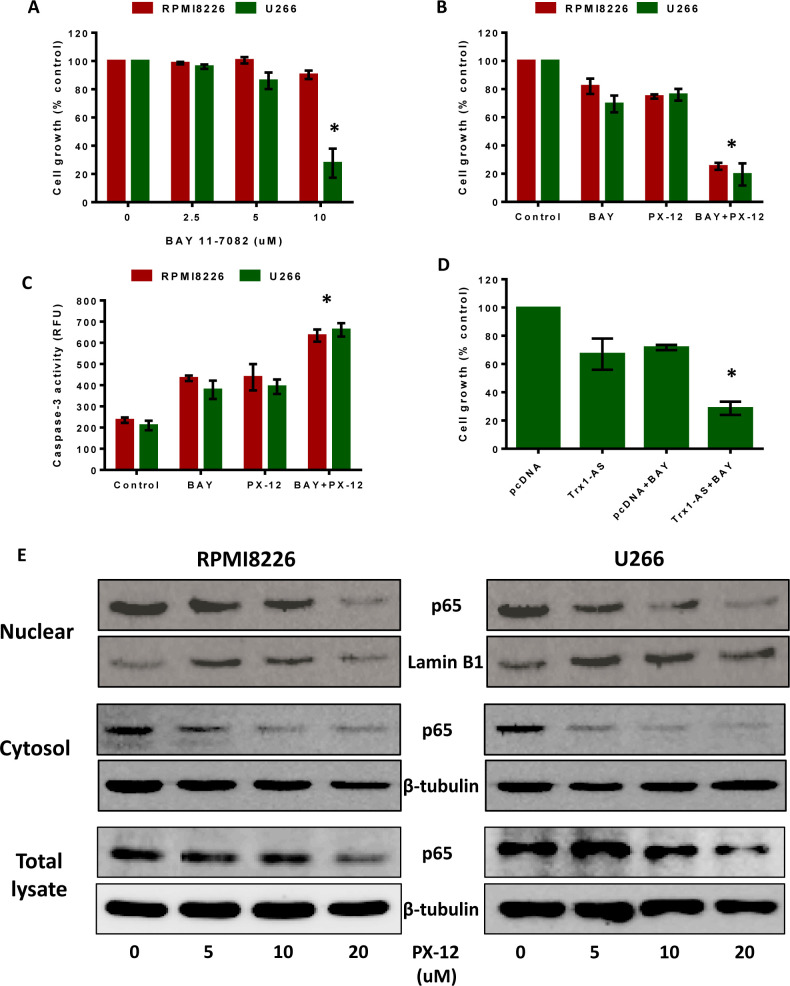
Trx1 inhibition sensitizes myeloma cells to NF-кβ inhibitors and decreases NF-кβ p65 protein expression **A.** RPMI8226 and U266 cells were treated with indicated concentrations of BAY 11-7082 for 24 hours and cell proliferation was assessed by MTT assays. **B. C.** RPMI8226 and U266 cells were treated with 2.5 μM PX-12 and 5 μM BAY 11-7082 alone or in combination for 24 hours and cell proliferation **B.** and caspase-3 activity were measured **C.**. **D.** U266 cells were transfected with Trx1-AS plasmid and pcDNA 3.1. 24 hours after transfections, cells were treated with or without 5 μM BAY 11-7082 for 24 hours and cell proliferation was assessed by MTT. Values indicated mean ± SEM of three independent experiments performed in triplicate. For caspase-3 activity assay (*n* = 3). One-way ANOVA followed by Tukey's post-test was employed. *, *P* < 0.05 (Compared to different treatment groups) **E.** RPMI8226 and U266 cells were treated with PX-12 (0-20 μM) for 24 hours. Nuclear, cytosolic, and total protein extracts were prepared and p65 protein expression was analyzed by western blot analysis. Lamin B1 (nuclear fractions) and β-tubulin (cytosolic fractions and total protein lysates) were used as loading controls. Western blots are the representative of three independent experiments.

Since MM cells have high NF-кβ activity, we tested the effect of the NF-кβ inhibitors, BAY 11-7082 [35] and curcumin [36], on MM cell proliferation using MTT assays. BAY 11-7082 had no effect on RPMI8226 cell proliferation, whereas U266 cell proliferation was inhibited only at a higher concentration (10 μM) (Figure 6A). Curcumin reduced RPMI8226 and U266 cell proliferation only at higher concentrations (10 μM and 20 μM) (Figure S3A). Our results also showed an increase in Trx1 protein levels in BAY 11-7082-treated RPMI8226 and U266 cells (Figure S4). Thus, increased Trx1 levels may be responsible for the decreased sensitivity of MM cells to NF-кβ inhibitors.

We then aimed to investigate the role of Trx1 in the resistance of MM cells to NF-кβ inhibitors. RPMI8226 and U266 cells were treated with a sub-lethal concentration of PX-12 (2.5 μM), with or without 5 μM BAY 11-7082, for 24 hours. Treatment with either PX-12 or BAY 11-7082 alone had no effect on cell proliferation. However, co-treatment of MM cells with both PX-12 and BAY 11-7082 significantly reduced RPMI8226 and U266 cell proliferation by 80% (Figure 6B). We then measured apoptosis in RPMI8226 and U266 cells treated with either PX-12 or BAY 11-7082 alone or in combination, which showed a significant increase in caspase-3 activity (Figure 6C) when cells were treated with PX-12 and BAY 11-7082 together. To confirm the results observed with PX-12, we used Trx1 knockdown using the Trx1-AS plasmid in combination with BAY 11-7082, which reduced U266 cell proliferation (Figure 6D). Similarly, treatment with either PX-12 or 5 μM curcumin alone had no effect on MM cell proliferation, but co-treatment with PX-12 and curcumin significantly reduced RPMI8226 and U266 proliferation by 40-45% (Figure S3B). Co-treatment with PX-12 and curcumin also significantly increased caspase-3 activity in RPMI8226 and U266 cells compared to treatment with either PX-12 or curcumin alone (Figure S3C).

Trx1 enhances the DNA biding activity of NF-кβ subunit p50 by reducing a cysteine at position 62 [29]. Based on this information, we investigated the effect of Trx1 inhibition on the expression and localization of NF-кβ subunit p65 in MM cells. Western blot results showed that PX-12 treatment significantly decreased cellular p65 protein levels, including in the nuclear and cytosolic fractions, in a concentration dependent manner in both MM cell lines (Figure 6E).

### Inhibition of Trx1 reduces proliferation and induces apoptosis of bortezomib-resistant MM cells

**Figure 7:**
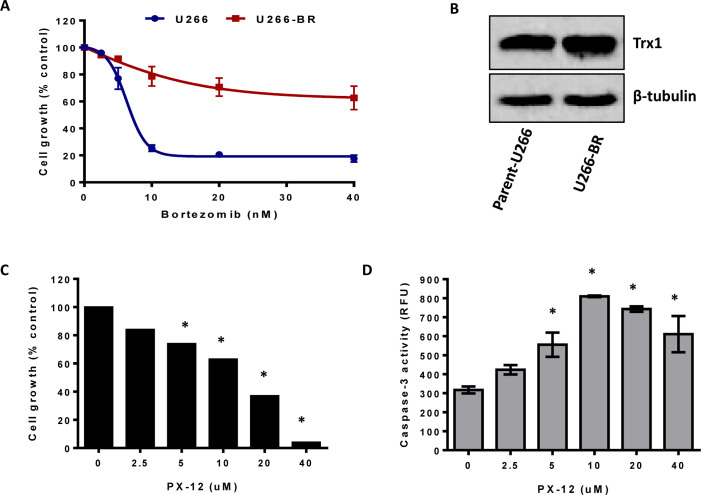
Inhibition of Trx1 reduces proliferation and induces apoptosis in bortezomib-resistant MM cells **A.** Parent-U266 and U266-BR cells were treated with bortezomib for 24 hours and cell proliferation was assessed by MTT assay. **B.** Trx1 protein expression in U266 and U266-BR cells was analyzed by western blot. β-tubulin was used as a loading control. **C. D.** U266-BR cells were treated with indicated concentrations of PX-12 for 24 hours. Cell proliferation was assessed by MTT assay **C.** and apoptosis was assessed by measuring caspase-3 activity **D.**. Values indicate mean ± SEM of three independent experiments performed in triplicate. For caspase-3 activity assay (*n* = 3). One-way ANOVA followed by Tukey's post-test was employed. *, *P* < 0.0001 (Compared to the control).

As MM cells acquire resistance to the bortezomib upon prolonged treatment resulting in disease relapse, we evaluated the effect of Trx1 inhibition on the growth and survival of drug-resistant myeloma cells. We first generated a bortezomib-resistant (BR) cell line (U266-BR) from the highly-sensitive parental U266 cell line (Figure 7A). Trx1 protein levels were observed to be higher in U266-BR cells compared to the U266 cells (Figure 7B). We next examined the effect of Trx1 inhibition on the survival of U266-BR cells. Treatment of U266-BR cells with PX-12 for 24 hours significantly inhibited their proliferation (Figure 7C). Moreover, PX-12 also increased caspase-3 activity in U266-BR cells indicating that cells have undergone apoptosis (Figure 7D).

## DISCUSSION

Despite the development of many anti-myeloma therapies, to date MM remains an incurable plasma cell malignancy [2-4] with drug resistance resulting in disease relapse. Therefore, development of new anti-myeloma therapies and strategies to treat drug-resistant MM patients is desperately needed. Many of the currently available anticancer drugs, including bortezomib, the most effective anti-myeloma drug, induce apoptosis by increasing the intracellular ROS levels and oxidative stress [37, 38]. However, cancer cells enhance their antioxidant capacity in response to increased oxidative stress to escape the toxic effect of drugs. Therefore, therapies that inhibit the activity of antioxidants to disrupt redox homeostasis may provide a more effective anti-myeloma therapy.

The role of redox proteins, Trx1 and TrxR1, in MM pathogenesis is largely unknown. Here, for the first time, we reported the cytoprotective role of Trx1 and TrxR1 in MM, and showed that disruption of intracellular redox homeostasis by inhibiting either Trx1 or TrxR1 reduces MM cell growth and survival. Our data showed that MM cells have higher intracellular ROS levels and increased Trx1 and TrxR1 expression levels compared to the normal PBMCs (Figure 1). We hypothesized that high Trx1 and TrxR1 protein levels may provide protection against increased oxidative stress and leads to MM cell survival and growth. As discussed earlier, PX-12 is an irreversible inhibitor of Trx1 and has been shown to inhibit tumor growth and lead to ROS-induced apoptosis in a variety of human cancers including acute myeloid leukemia [32], colorectal cancer [39], and lung cancer [33, 40]. PX-12 has been tested in two independent clinical studies, one in advanced gastrointestinal cancer and another in patients with advanced pancreatic cancer [41, 42]. Although no significant anti-tumor activity of PX-12 was observed in both clinical studies, the Trx1 pathway was suggested as an effective therapeutic target in cancer. Auranofin, a TrxR1 inhibitor, leads to oxidative stress-induced lethality and apoptosis in cancers including chronic myeloid leukemia [19], chronic lymphocytic leukemia [43], prostate cancer [44], breast cancer [45], and acute leukemia [46]. Auranofin is currently in a phase II clinical trial for chronic lymphocytic leukemia and adenosquamous cell lung cancer patients. Because of the well documented anti-tumor activity of PX-12 and auranofin, we used these drugs, along with knockdown of Trx1 and TrxR1, as tools to study the cytoprotective role of Trx1 and TrxR1 in MM. In line with other studies reporting the cytoprotective role of Trx1 and TrxR1 [17, 47], we showed that both drug-induced inhibition and specific knockdown of Trx1 and TrxR1 reduced MM cell proliferation, induced cell death, and apoptosis (Figures 2 and 4). Inhibition of Trx1 and TrxR1 using PX-12 and auranofin, respectively, had little or no cytotoxic effect on non-cancerous PBMCs isolated from healthy individuals (Figures 2 and 4). Hence, our study suggests that both Trx1 and TrxR1 can be used as effective therapeutic targets to treat MM.

The Trx system maintains the intracellular redox homeostasis by scavenging ROS and regulating various redox enzymes [9-11]. Inhibition of the Trx system increases intracellular oxidative stress [48, 49]. We showed that Trx1 and TrxR1 inhibition increased intracellular ROS levels and induced MM cell apoptosis (Figures 3 and 5). Treatment of cells with antioxidants, such as NAC or ascorbic acid, has reduced the amount of ROS generated in response to Trx inhibition, and rescued cells from undergoing apoptosis.

Overexpression of Trx1 and TrxR1 has been linked to the aggressive phenotype of many cancer types, including aggressive invasive mammary carcinomas and advanced malignant melanoma [15]. Trx1 overexpression has been shown to promote breast cancer cell invasion by stimulating MMP-9 expression. Inhibition of Trx1 activity has been shown to decrease MMP-9 expression via suppression of NF-кβ activity and expression of NF-кβ subunits, p50 and p65, in breast cancer cells [50]. We showed that inhibition of Trx1 resulted in decreased protein levels of NF-κβ subunit p65 in MM cells. Moreover, Trx1 inhibition has been shown to reduce the clonogenic activity in diffuse large B-cell lymphoma [17]. Our findings showed that drugs inhibiting either Trx1 or TrxR1 decreased the clonogenic activity in MM cells. Interestingly, cells treated with 10 μM PX-12 resulted in the formation of larger colonies compared to the non-treated cells, although 10 μM PX-12 treatment reduced the number of colonies in both cell lines. MM cell lines, including RPMI8226 and U266, and primary MM tumor cells have been shown to possess a small side population (SP) of the cells that exhibit more clonogenic and tumorigenic capacity [51]. These SP cells possess stem cell-like features and express high levels of ABC transporter family (ABCB1 and ABCG2), which are responsible for SP cell's drug-resistance [34, 52-54]. Moreover, these SP cells have more clonogenic potential and the colonies formed by SP cells are larger than the colonies formed by non-SP cells [51, 55]. Based on this information, we postulated that the larger colonies observed in the samples treated with 10 μM PX-12 may have formed from the SP cells present in RPMI8226 and U266 cells, which may be resistant to PX-12. Thus, an upregulated Trx system is linked not just to cancer cell proliferation, invasion, metastasis, but also to clonogenic activity.

This study, for the first time, highlights the cytoprotective role of Trx1 in the resistance of MM cells to NF-кβ inhibitors. We showed that Trx1-inhibited MM cells become more sensitive towards the NF-кβ inhibitors BAY 11-7082 and curcumin. BAY 11-7082 had no effect on RPMI8226 cell growth at any concentrations, while only higher concentrations of BAY 11-7082 reduced U266 cell growth (Figure 6), and Trx1 protein levels were markedly increased in response to BAY 11-7082 treatment in both MM cell lines. Similarly, only higher concentrations of curcumin reduced RPMI8226 and U266 cell proliferation (Figure S3). Elevated levels of Trx1 have been implicated in the resistance of cancer cells to several chemotherapeutic agents including cisplatin, docetaxel, and doxycycline [16, 56]. Inhibition of Trx1 has been shown to reverse the drug-resistance in many cancer types [17]. We hypothesized that high levels of Trx1 observed in myeloma cells may exert cytoprotection against NF-кβ inhibitors. Our data showed that inhibition of Trx1 using a sub-lethal concentration of PX-12 (2.5 μM) sensitized MM cells to undergo apoptosis in response to BAY 11-7082 (Figure 6) or curcumin (Figure S3). The amount of cell death and caspase-3 activity was higher in cells treated with PX-12 and BAY 11-7082 or curcumin together compared to the treatment with either PX-12 or BAY 11-7082 or curcumin alone. Specific knockdown of Trx1 using the antisense Trx1 plasmid produced similar results (Figure 6). Thus, Trx1 inhibition sensitized MM cells to NF-кβ inhibitors suggesting that increased Trx1 levels may be responsible for the resistance of MM cells to NF-кβ-targeted therapies. In addition, we showed that Trx1 inhibition resulted in decreased protein levels of NF-кβ subunit p65 in MM cells. Therefore inhibiting Trx1 in conjunction with the NF-кβ pathway may lead to a better anti-myeloma therapy.

In recent years, many anti-myeloma drugs, including bortezomib, thalidomide, and dexamethasone, have been developed [57, 58]. Bortezomib, a proteasome inhibitor, has been shown to inhibit proliferation and induce apoptosis of human MM cell lines and primary MM patient cells, without any significant cytotoxic effect on non-cancerous PBMCs [59]. Upon prolonged treatment with these drugs MM cells acquire resistance, resulting in relapse [28, 60]. This study highlighted a novel strategy to treat drug-resistant MM by inhibiting Trx1. We showed that the Trx1 inhibiting drug PX-12 induced apoptosis in bortezomib-resistant MM cells. These bortezomib-resistant U266 cells had higher Trx1 protein levels compared to the parent U266 cells (Figure 7B). In line with other studies indicating the role of Trx1 in chemoresistance [16, 17, 56], our results showed that Trx1 inhibition sensitized bortezomib-resistant U266 cells to undergo apoptosis (Figure 7D). Our findings complement a study that showed that inhibition of another major antioxidant system, the glutathione system, strongly enhances bortezomib-toxicity in MM [61]. Inhibition of Trx1, might not only offer a novel approach to treat newly diagnosed MM patients, but also those with drug-resistant relapsed/refractory MM.

In conclusion, this research confirms that Trx1 inhibition results in ROS-induced apoptosis in MM cells, sensitizes MM cells to the NF-кβ inhibitors, and also induces apoptosis in bortezomib-resistant myeloma cells. Thus, Trx1 may be an effective therapeutic target to treat newly diagnosed and relapsed/refractory MM patients. This research provides an important insight into the cytoprotective role of Trx1 and TrxR1 in MM cell survival and growth, which may lead to the development of new, more effective approaches to treat MM, which is currently incurable. This study recommends considering Trx1 and TrxR1 as molecular targets for inhibition, which holds promising anti-myeloma therapy.

## MATERIALS AND METHODS

### Cells and reagents

Human multiple myeloma cell lines (RPMI8226, U266) were obtained from Dr. Slavica Vuckovic (QIMR Berghofer Medical Research Institute). Human peripheral blood mononuclear cells (PBMCs) were isolated from the whole blood of healthy volunteers and were collected under the ethical approval BPS/08/14/HREC. The cells were cultured in RPMI-1640 medium (Gibco, VIC, Australia) containing 10% (V/V) fetal bovine serum (FBS) (Gibco), 200 mM L-glutamine (Invitrogen, VIC, Australia), and 100 U/ml penicillin and 100 μg/ml streptomycin (Invitrogen). The Trx1 antibody (5G8) is a mouse monoclonal antibody generated against recombinant human thioredoxin [62]. The monoclonal anti-TrxR1 antibody was purchased from R&D systems (MN, USA). The polyclonal anti-NF-кβ p65 antibody was purchased from GeneTex (CA, USA) and anti-β-tubulin antibody was purchased from Abcam. The Trx1 inhibitor PX-12 (1-methylpropyl 2-imidazolyl disulfide) was purchased from Cayman Chemicals (MI, USA). The NF-κβ inhibitors BAY 11-7082 and curcumin were purchased from Cayman Chemicals and Sigma. The TrxR1 validated small interfering RNA (siRNA) and control siRNA were purchased from Santa Cruz Biotechnology (TX, USA). The Trx1-anti sense plasmid was made by reversing the orientation of the Trx insert in pcDNA3.1 (Invitrogen) of a pTrx-WT plasmid previously described [63].

### Database

Gene expression data was obtained from the gene expression profile arrays in patient myeloma cells at different stages of the disease and healthy cells deposited in the gene expression omnibus database GSE6477.

### Western blot analysis

Western blot analysis was performed as described previously [62]. Whole cell extracts were prepared using 0.5% (v/v) Nonidet-40 cell lysis buffer. Nuclear and cytosolic fractions were prepared using Nuclear Protein Extraction Kit (Cayman Chemicals) according to the manufacturer's guidelines. Protein samples were solubilized with 5% SDS (w/v) sample buffer and electrophoresed on polyacrylamide gel. Proteins were transferred onto a polyvinylidene difluoride membrane and were probed with various specific antibodies (Trx1, TrxR1, p65, β-tubulin) and the appropriate horseradish peroxide-conjugated secondary antibodies.

### Measurement of intracellular ROS generation

A dicholorofluorescein (DCF) assay was used to determine cellular ROS generation in MM cells and control PBMCs as described previously [64]. Briefly, either 1 × 10^6^ or 1 × 10^5^ of treated or untreated cells were washed with PBS and incubated with 10 μM H_2_DCFDA (Molecular probes, CA, USA), a redox sensitive cell permeable dye, for 15 minutes. Cells were then transferred to black-walled clear-bottom 96-wells plate in triplicate and assessed for H_2_DCFDA oxidation using a SpectraMax fluorescence plate reader (Molecular Devices). Data were analyzed using SoftMax Pro software (Bio-strategy). The fluorescence intensity measuring the oxidation of H_2_DCFDA by ROS represents the amount of intracellular ROS generation.

### Cell proliferation assay

RPMI8226, U266, and PBMCs (0.5 × 10^6^ cells per well of 24-wells plate) were treated with appropriate inhibitors for 24 hours. After which, cell proliferation was determined using MTT solution (Sigma, NSW, Australia).

### Cell viability measurement

Cell viability was measured by the Trypan blue exclusion method. Treated and untreated cells were stained with Trypan blue and viable cells were counted using an inverted light microscope.

### Caspase-3 activity assay

Caspase-3 activity within the treated and untreated MM cell lines and PBMCs was determined as described previously following the cleavage of Ac-DEVD-AMC (Enzo Life Sciences, NY, USA), a caspase-3 substrate [65]. Briefly, treated or untreated cells (0.5 × 10^6^ cells) were pelleted, washed with PBS, re-suspended in 10-15 μl of PBS, and transferred to black-walled 96-wells plate. 90 μl of caspase assay buffer (5 mM dithiothreitol, 100 mM HEPES, 10% (w/v) sucrose, 0.1% NP-40 at pH 7.25) containing 50 μM Ac-DEVD-AMC was added to the samples and the amount of AMC cleaved by caspase-3 was measured at 37°C by measuring the fluorescence at excitation wavelength of 370 nm and emission of 445 nm using SpectraMax plate reader.

### Transient transfections

Cells (1.5 × 10^6^ per well) were transfected using Amaxa Nucleofector (T-001 program) using equivalent molar concentrations of siRNAs (final concentrations of 30 nmol/L). Empty vector, pcDNA 3.1, and Trx1-anti sense plasmids (2 μg) were transfected using the same protocol. Transfected cells were incubated for 24 hours before the indicated treatments.

### Establishment of bortezomib-resistant cell lines

Bortezomib-resistant (BR) MM cell line (U266-BR) was generated from a parental cell line (U266) by multistep exposure of the cells to increasing doses of bortezomib (40 nM) for 12 weeks. Briefly, cells were initially cultured in a low drug concentration for 1 week followed by 1 week in a drug-free medium for stabilization. Cells were exposed to increasing concentrations of bortezomib for 1 week followed by 1 week in a drug-free medium before the next bortezomib exposure for 12 weeks, and subsequently cells became resistant to bortezomib. The resistant cells were expanded in a drug-free medium. Cells were tested for drug resistance before any further studies.

### Methylcellulose clonogenic assay

The colony formation assay in methylcellulose (R&D systems) was performed according to the manufacturer's instructions. Briefly, treated and untreated MM cells were plated at the density of 1000 cells/ml in 1.1ml volume in 35 mm petri dishes. Each plate contained RPMI-1640 media consisting of 1.4% methylcellulose, 25% (v/v) FBS, 100 U/ml penicillin and 100 μg/ml streptomycin, and 1% (w/v) BSA. Plates were incubated at 37°C, 5% CO_2_ for 8-10 days. To observe colonies, cells were stained with 0.5 mg/ml MTT (Sigma), 500 μl/plate [66]. After incubating the plates for 1 hour at 37°C, colonies containing more than 30 cells were counted using an inverted light microscope. Colonies were photographed using a Tucsen ix30 camera and TSview software (Tucsen, China).

### Statistical analysis

All values are presented as mean ± SEM. Data was analyzed using the software GraphPad Prism 6 (GraphPad Software, CA, USA). Statistical significance was determined by One-way ANOVA followed by Tukey's post-test and Two-way ANOVA followed by Sidak's post-test. *P* < 0.05 was considered significant.

## SUPPLEMENTARY MATERIALS, FIGURES


