# Correction: Inhibition of thioredoxin 1 leads to apoptosis in drug-resistant multiple myeloma

**DOI:** 10.18632/oncotarget.27444

**Published:** 2021-04-27

**Authors:** Prahlad V. Raninga, Giovanna Di Trapani, Slavica Vuckovic, Maneet Bhatia, Kathryn F. Tonissen

**Affiliations:** ^1^ School of Natural Sciences, Griffith University, Nathan, QLD, Australia; ^2^ Eskitis Institute for Drug Discovery, Griffith University, Nathan, QLD, Australia; ^3^ QIMR Berghofer Medical Research Institute, Brisbane, QLD, Australia

**This article has been corrected:** Due to errors in image selection, Figure 3A inadvertently included copies of the same panel within the same figure. This is in respect to the 5 μM PX-12 treatment of the cell lines, where 2 images have been repeated between the RPMI8226 and U266 cells. We have now repeated the entire experiment and analyzed the data. The corrected Figure 3, obtained via the new experiment, is shown below. The authors declare that these corrections do not change the results or conclusions of this paper.

Original article: Oncotarget. 2015; 6:15410–15424. 15410-15424. https://doi.org/10.18632/oncotarget.3795

**Figure 3:**
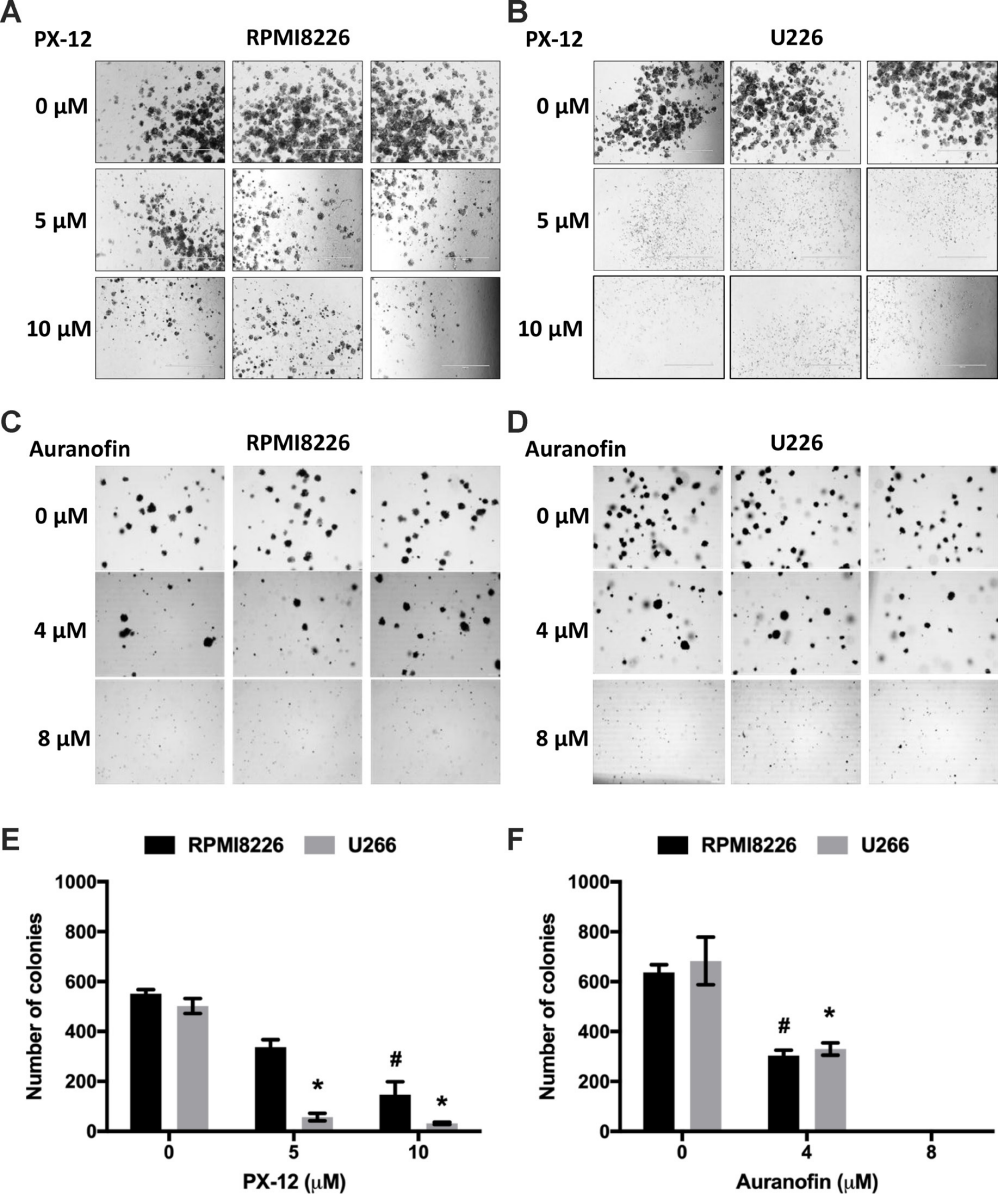
TrxR1 and Trx1 inhibition reduces clonogenic activity of MM cells. (**A**, **B**) Methylcellulose colony formation assay of RPMI8226 (A) and U266 (B) cells treated with 5 µM and 10 µM PX-12. (**C**, **D**) Methylcellulose colony formation assay of RPMI8226 (C) and U266 (D) cells treated with 4 µM and 8 µM auranofin. (**E**) Number of colonies formed after PX-12 treatment of RPMI8226 and U266 cells. (**F**) Number of colonies formed after auranofin treatment of RPMI8226 and U266 cells. Values indicate mean ± SEM of three independent experiments performed in duplicate. One-way ANOVA followed by Tukey’s post-test was employed. * (U266), # (RPMI8226), P < 0.05.

