# Supplementary material for: Inhibition of thioredoxin 1 leads to apoptosis in drug-resistant multiple myeloma
Source: Oncotarget. 2015 Apr 12;6(17):15410–24. doi: 10.18632/oncotarget.3795 (PMC4558160; doi:10.18632/oncotarget.3795)
Supplement: Supplementary file 1 [file oncotarget-06-15410-s001.pdf]

Inhibition of thioredoxin 1 leads to apoptosis in drug-resistant multiple myeloma

Supplementary Material

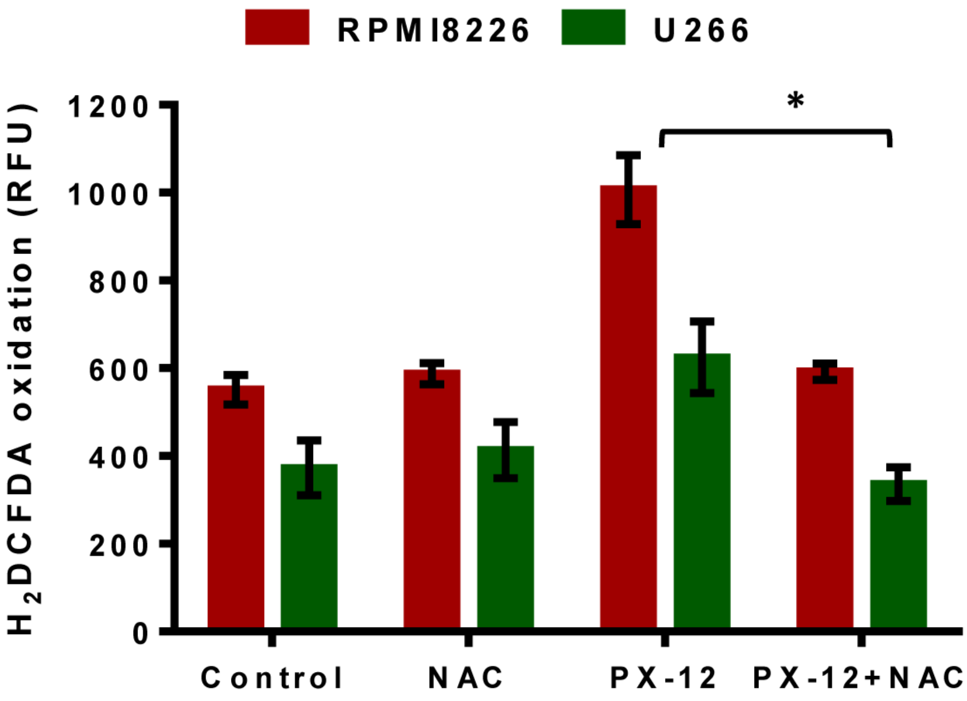

Figure S1: N-acetyl cysteine scavenges the increased intracellular ROS generated in response to Trx1 inhibition in MM cells. RPMI8226 and U266 cells were treated with 5 µM PX-12 alone or in combination with 5 nM NAC followed by assessing H_2DCFDA oxidation. One-way ANOVA followed by Tukey's post-test were employed. *, $P < 0.05$ Values indicate mean ± SEM of three independent experiments performed in triplicate.

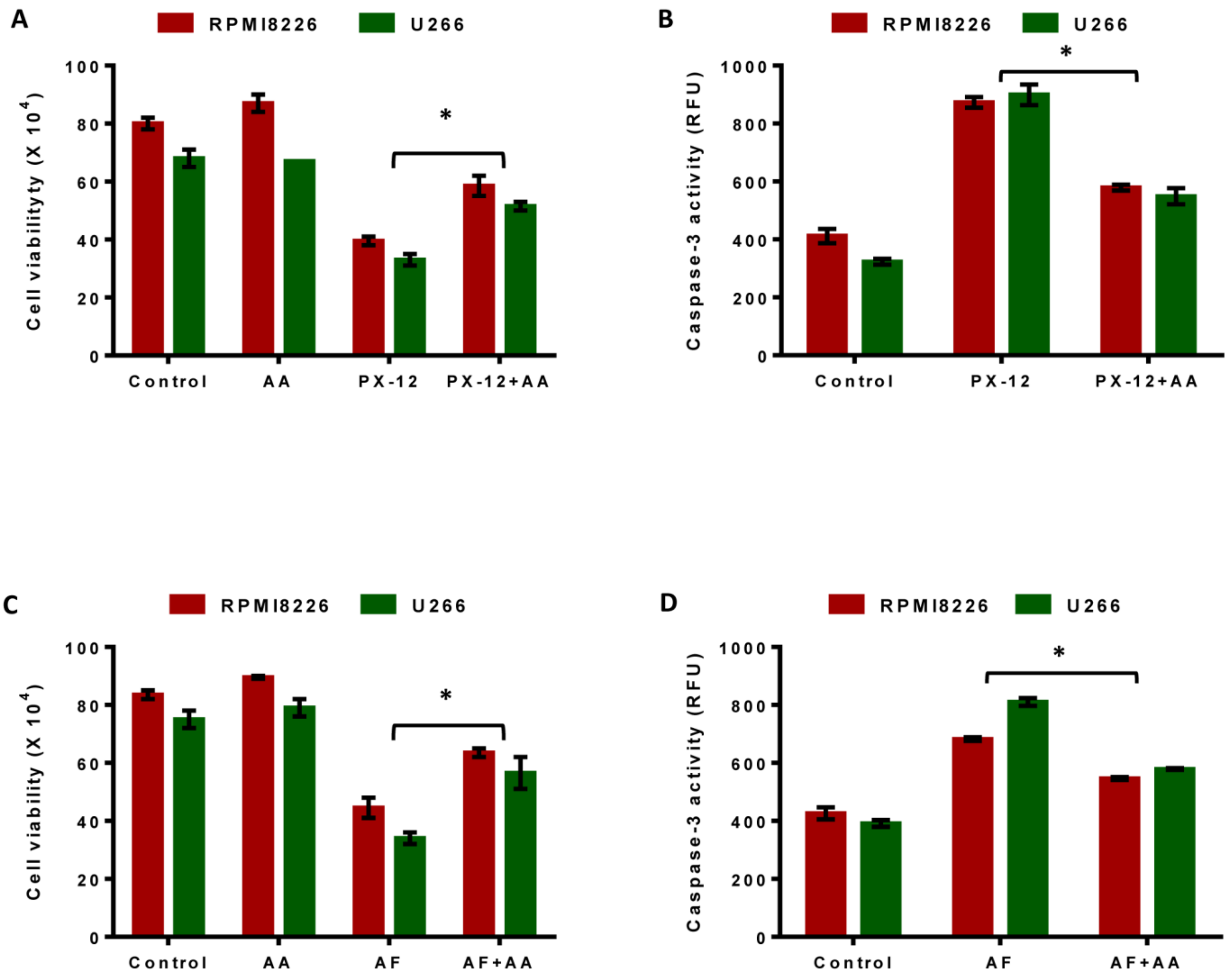

Figure S2: The antioxidant, ascorbic acid, rescues MM cells from undergoing apoptosis in response to Trx1 and TrxR1 inhibition. A, B, RPMI8226 and U266 cells were treated with 5 µM PX-12 alone or in combination with 100 µM ascorbic acid (AA) for 24 hours followed by measuring cell viability using Trypan blue exclusion (A), and examination of apoptosis by measuring caspase-3 activity (B). **C, D,** RPMI8226 and U266 cells were treated with 2 µM auranofin alone or in combination with 100 µM AA for 24 hours followed by measuring cell viability using Trypan blue exclusion (C) and examination of apoptosis by measuring caspase-3 activity (D). One-way ANOVA followed by Tukey's post-test was employed. *, $P < 0.05$. Values indicate mean ± SEM of three independent experiments performed in triplicate. For caspase-3 activity assay (n = 3).

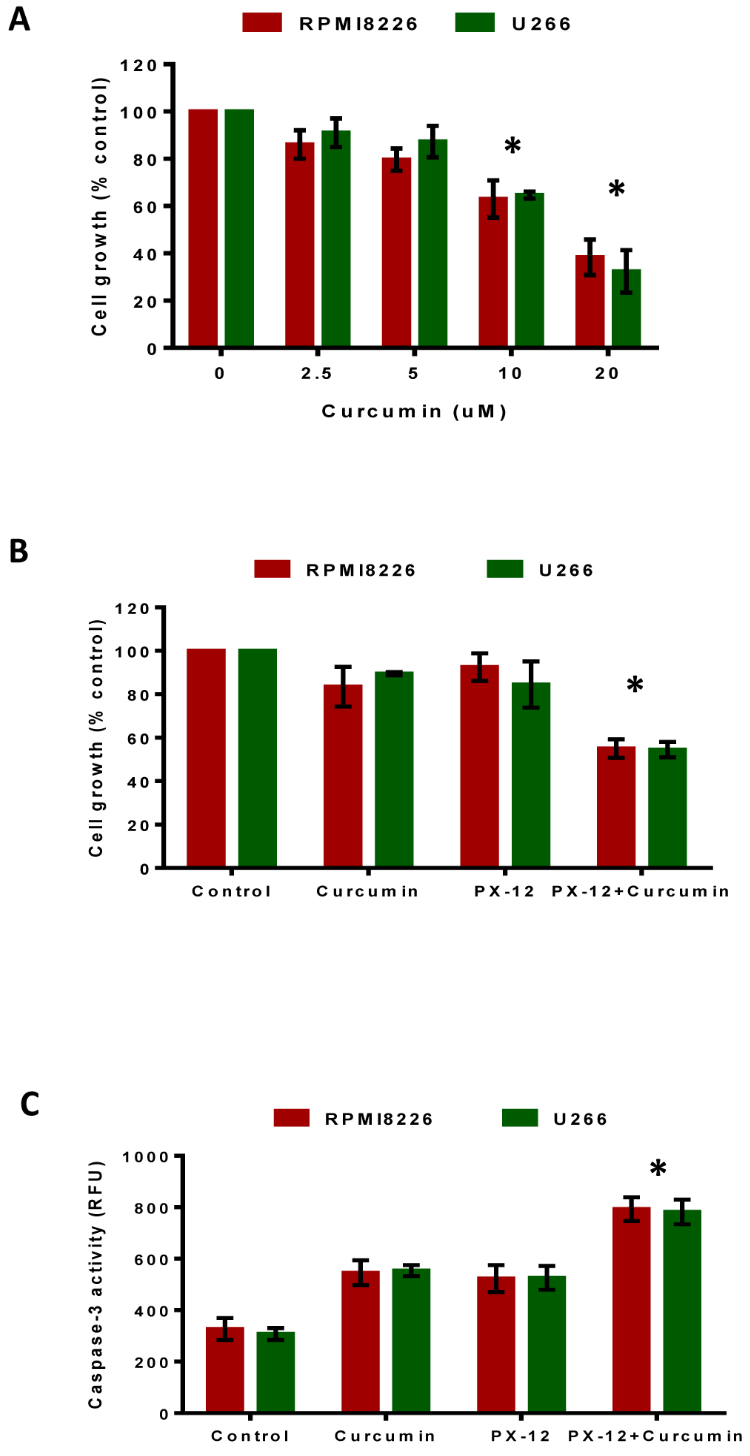

Figure S3: Trx1 inhibition sensitizes MM cells to the NF-κβ inhibitor, curcumin. A, RPMI8226 and U266 cells were treated with indicated concentrations of curcumin for 24 hours and cell proliferation was assessed by MTT assays. B, C, RPMI8226 and U266 cells were treated with 2.5 µM PX-12 and 5 µM curcumin alone or in combination for 24 hours and cell proliferation (B) and caspase-3 activity were measured (C). Values indicated mean ± SEM of three independent experiments performed in triplicate. For caspase-3 activity assay ($n = 3$). One-way ANOVA followed by Tukey's post-test was employed. *, $P < 0.05$ (Compared to different treatment groups)

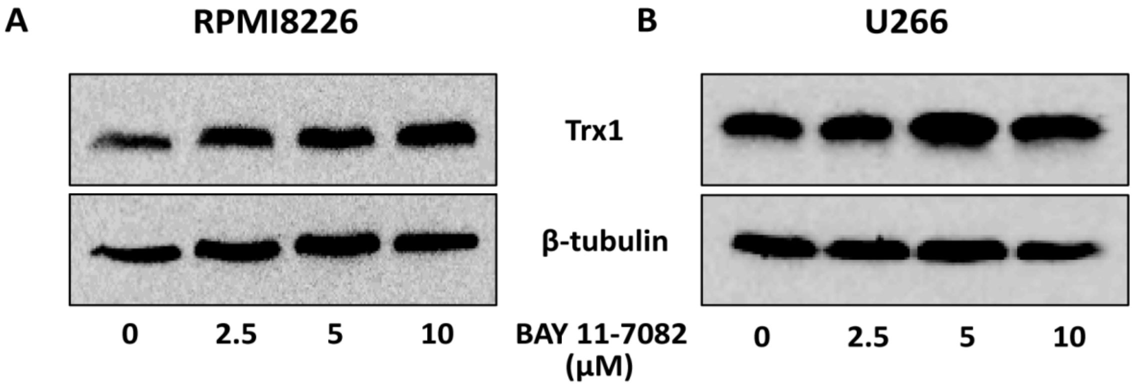

Figure S4: Inhibition of NF-κβ upregulates Trx1 protein levels in MM cells. A, RPMI8226, and **B,** U266 cells were treated with indicated concentrations of NF-κβ inhibitor, BAY 11-7082 for 24 hours. Trx1 protein expression was analyzed by western blot analysis. β-tubulin was used as a loading control. Western blots are the representative of three independent experiments.
